# In‐Silico Trial of Same‐Day Simulation‐Free Spatially Fractionated Adaptive Radiotherapy (SF^2^‐ART)

**DOI:** 10.1002/acm2.70517

**Published:** 2026-02-16

**Authors:** Dennis N. Stanley, Alyssa R. Birchmeier, Carlos E. Cardenas, Richard A. Popple, Natalie Viscariello, Joel A. Pogue, Courtney B. Stanley, Mehran Yusuf, Michael Soike, Samuel R. Marcrom, Joseph Harms

**Affiliations:** ^1^ Department of Radiation Oncology University of Alabama at Birmingham Birmingham Alabama USA; ^2^ Department of Radiation Oncology Washington University School of Medicine St. Louis Missouri USA

**Keywords:** Online Adaptive RT, Simulation Free, Spatially Fractionated, Virtual Clinical Study, CT Guided Adaptive RT (CTgART)

## Abstract

**Purpose:**

Here we present an in‐silico trial of the feasibility and deliverability of a same‐day, simulation‐free workflow for spatially fractionated adaptive radiotherapy (SF^2^‐ART) using the Varian Ethos platform.

**Methods and Materials:**

Ten patients (five thoracic and five extremity), previously treated with spatially fractionated radiotherapy (SFRT), were selected for this in silico trial. A two‐phase regimen was simulated: Phase 1 delivered 12 Gy SBRT to a uniform, predefined high‐dose lattice and 4 Gy to the gross tumor volume (GTV); Phase 2 consisted of 4 additional fractions of 4 Gy to the GTV. The planning workflow was performed entirely in silico, without the need for CT simulation. High‐dose sphere matrices were generated with a custom script and aligned to physician‐defined GTVs. Adaptive plans were created based on CBCT anatomy and evaluated for dosimetric quality, deliverability, and workflow timing. Comparisons were made to the original clinically delivered plans. Mobius3D was used for secondary dose verification.

**Results:**

All 10 cases were successfully replanned using the SF^2^‐ART workflow without simulation‐based imaging. The entire planning and treatment process, including consultation, contouring, adaptive plan generation, and QA, was performed within a single session. Dosimetric comparisons showed minimal differences between simulation‐free adaptive and clinically delivered plans, with target coverage and normal tissue constraints maintained across all cases. Median total estimated workflow time was 166.4 min.

**Conclusions:**

This in‐silico study demonstrates the feasibility of delivering spatially fractionated SBRT without simulation CT using a same‐day adaptive workflow. The SF^2^‐ART approach may enable rapid, high‐quality treatment for patients with urgent or symptomatic presentations and supports future prospective clinical implementation.

## 1 INTRODUCTION

Spatially fractionated radiotherapy (SFRT) is increasingly used to treat large, radioresistant tumors that are difficult to manage with conventional techniques.^1^, ^2^ By delivering high doses to very small regions within a tumor, SFRT aims to improve tumor control by leveraging biological responses like vascular disruption, immune stimulation, and bystander effects.^3^ Clinically, SFRT is often employed in palliative or urgent settings, where patients present with symptomatic, bulky disease that compromises quality of life. In these cases, rapid treatment initiation can be critical, not only to alleviate symptoms but also to prevent further tumor progression.^4^

With the recent expansion of online adaptive radiotherapy (ART) in clinical practice, there is growing interest in using ART for time‐sensitive cases, as ART can account for rapid anatomical changes and streamline the treatment process.^5^ Online ART involves real‐time plan modification based on daily imaging, allowing clinicians to adjust treatment volumes and optimize dose distributions immediately prior to delivery.^5^, ^6^ This approach helps maintain plan quality in the presence of patient changes between consultation, simulation, and treatment delivery.

However, traditional planning workflows, which consist of CT simulation, physician contouring, and iterative plan development, commonly take several days. At our institution, the average interval from consultation to first SFRT fraction is approximately 11 days, underscoring how standard multi‐day workflows can delay care for patients presenting with bulky, symptomatic tumors. For patients with rapidly progressing disease, delays may lead to changes in anatomy or loss of treatment opportunity altogether.^7^ To address these limitations, recent studies have demonstrated the feasibility of simulation‐free ART workflows using high‐quality diagnostic CTs or CBCT scans acquired directly on the treatment unit.^4^, ^8^, ^9^, ^10^, ^11^ These approaches have shown promise in both palliative and definitive contexts, enabling planning and treatment to occur within the same day.^12^

In this study, we conduct an in‐silico study evaluating the feasibility of a simulation‐free, spatially fractionated adaptive radiotherapy (SF^2^‐ART) workflow using the Varian Ethos platform. The proposed approach leverages either a high‐quality CBCT or suitable diagnostic imaging, combined with a templated planning and delivery process. We hypothesize that SF^2^‐ART can be safely and effectively delivered without the need for simulation‐based CT, enabling a complete consult‐to‐treatment workflow within a single session, ideally in under three hours.

## 2 METHODS

### 2.1 Patient selection and in silico treatment platform

This IRB‐approved (#IRB‐300014635), retrospective in silico feasibility study included the 10 most recently treated SFRT patients at our institution. The cohort consisted of five patients with thoracic tumors and five with extremity tumors. Each patient previously received a two‐phase treatment course: Phase 1 consisted of a single‐fraction SBRT delivering 12 Gy to a high‐dose spherical lattice matrix and 4 Gy to the gross tumor volume (GTV), followed by Phase 2 with 16 Gy in four additional fractions of 4 Gy prescribed to PTV_Low (a uniform 3–5 mm expansion of the GTV), totaling 20 Gy to the GTV/PTV_Low target region.

For this study, all cases were replanned and evaluated using a proposed simulation‐free workflow implemented on the Varian Ethos platform^13^ (Varian Medical Systems, Palo Alto, CA, Version 2.1) in conjunction with the Eclipse treatment planning system (Varian Medical Systems, Palo Alto, CA, Version 18.1). The analysis was conducted entirely in silico; both treatment planning and delivery were simulated using an Ethos emulator, a virtual platform that replicates the clinical adaptive planning and treatment environment.

Successful implementation of the SF^2^‐ART workflow was defined as generation of an adaptive plan that met all predefined clinical and dosimetric objectives, was completed in under four hours, utilized either a diagnostic CT or image‐only CBCT for planning, and was confirmed as technically deliverable using standard QA tools. A clinically acceptable plan was defined as one that met physician‐reviewed target coverage requirements and normal tissue constraints. Specifically, for PTV_High, plans were required to achieve V12Gy ≥ 90% and D0.03cc ≤ 15 Gy. For PTV_Low, dosimetric objectives included V4Gy ≥ 95%, D0.03cc ≤ 12 Gy, and Dmean between 3.5 and 5.0 Gy. Normal tissue constraints for organs‐at‐risk (OARs) were evaluated on a case‐by‐case basis and followed standard clinical practice for the relevant anatomical site. Final plan deliverability was confirmed through secondary dose calculation using Mobius3D with a 3D gamma pass rate >90% under 3%/2 mm criteria and a 10% threshold, as well as monitor unit (MU) verification and an independent technical review by a qualified medical physicist.

### 2.2 Pre‐plan workflow

**FIGURE 1:**
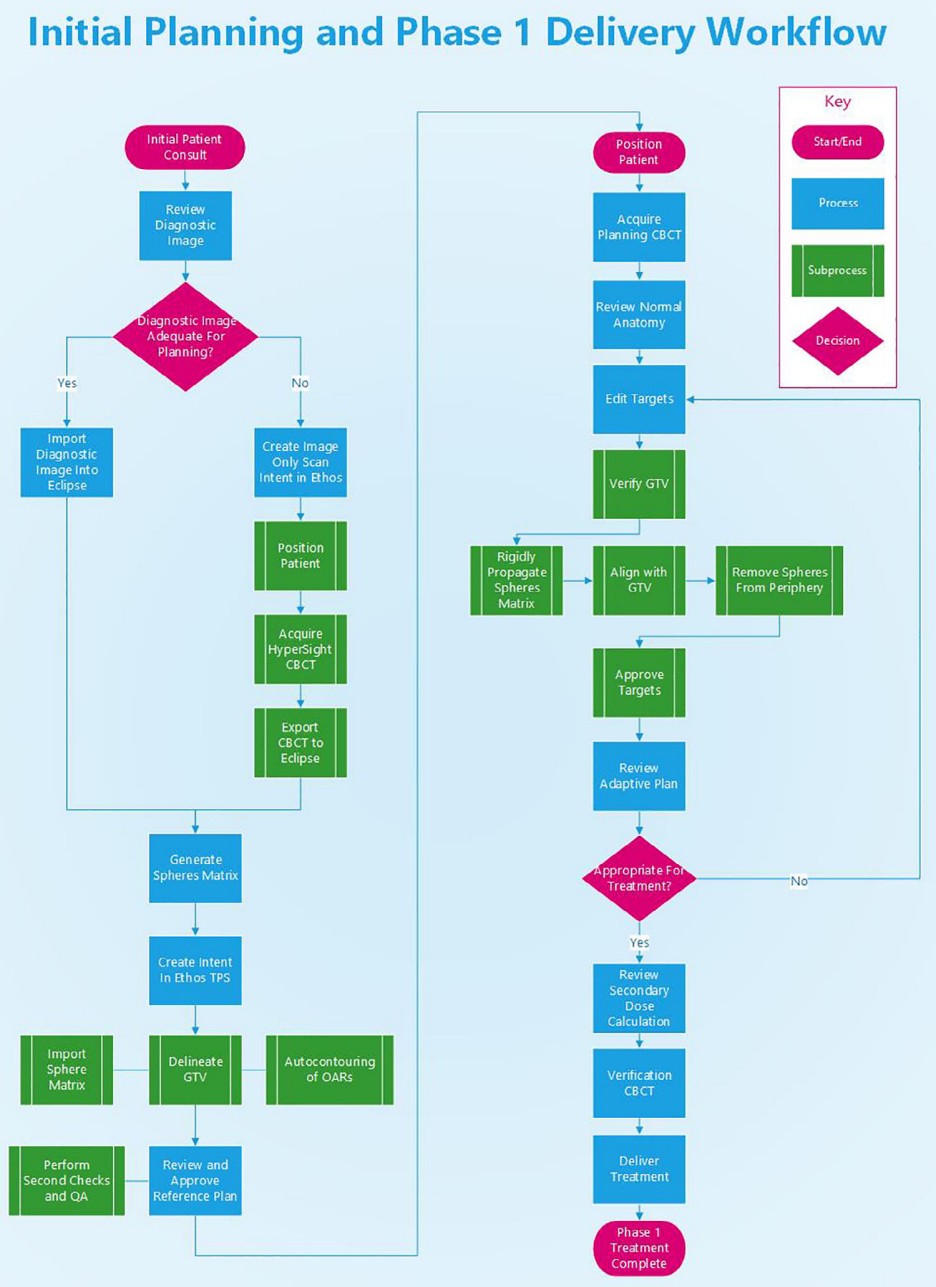
Workflow for same‐day simulation‐free SF^2^‐ART planning and Phase 1 treatment. The process includes image acquisition (diagnostic CT or HyperSight CBCT), templated lattice plan creation, adaptive contouring and optimization, and on‐couch treatment delivery with secondary dose verification.

Each case began with a review of available imaging to assess suitability for treatment planning. In the proposed clinical workflow, this step would occur immediately after patient consultation. Recent diagnostic CT scans were reviewed for planning purposes, with suitability determined based on clinical judgment. Factors evaluated included full anatomical coverage, appropriate field‐of‐view (FOV), patient positioning, and overall image quality. If a diagnostic CT was unavailable or inadequate, a dedicated image‐only scan could be performed on the Ethos system using HyperSight CBCT. This scan, acquired with the patient in treatment position, serves as the planning image. For this retrospective study, previously acquired CBCTs were used in place of prospective image‐only scans for half of the evaluated patients (2 Thorax and 3 Extremities). The complete proposed planning and delivery workflow is illustrated in Figure 1.

A lattice of high‐dose spheres was generated using an in‐house Eclipse Scripting Application Programming Interface (ESAPI) script. The script modifies the Eclipse‐generated structure set to insert a new lattice structure that consists of 1 cm diameter spheres arranged in 3D grid with a minimum inter‐sphere spacing set by the user. The spheres are positioned relative to a defined center point, following the principles outlined by Duriseti et al.^3^ The matrix size was chosen based on a rough over estimation of the maximum length of the GTV to be treated. For the 10 evaluated cases, lattice matrix volume of 20 × 20 × 20 cm were used in eight cases; in two cases, this was expanded to 30 × 30 × 30 cm to ensure full target coverage. Sphere size and spacing were held constant across all patients, with each sphere measuring 1 cm in diameter and spaced 2 cm center‐to‐center as described by Duriseti et al.^3^

Once generated, the planning image and modified structure set were imported into Ethos for reference plan construction. All reference plans were created using a standardized Ethos planning template. Any clinically relevant normal tissue structures and OARs were contoured via Ethos’ auto‐segmentation tools.^14^ Optimization was performed using the Ethos 2.0 Intelligent Optimization Engine (IOE),^15^ with planning priorities focused on limiting hotspots to below 15 Gy (125%) and minimizing dose spill outside the high‐dose spheres and GTV. Table 1 summarizes the standard structures, derivations, and any associated optimization objectives.

**TABLE 1:** Planning structures, derivation methods, and associated optimization objectives used in the SF^2^‐ART workflow.

Structure name	Derivation method	Optimization objectives
GTV	Physician‐delineated on planning image	None (not used for optimization)
_SpheresMatrix	Uniform 3D lattice of spheres	None (not used for optimization)
PTV_High	Overlap of _SpheresMatrix and 1 cm retraction of GTV	V12.00 Gy ≥ 90% Dmax (0.03)cc ≤ 15.00 Gy
PTV_Low	GTV structure expanded uniformly by 3–5 mm	D95% ≥ 4.00 Gy Dmax (0.03)cc ≤ 12.00 Gy Dmean ≥3.50 Gy, ≤ 5.50 Gy
Normal tissue ring	(PTV_Low + 3 cm)—PTV_Low	Dmax (0.03)cc ≤ 4.00 Gy
OARs	Auto‐segmented in Ethos TPS	Institutional standard clinical constraints; reviewed/adjusted as needed
Body	Derived from external contour	None (not used for optimization)

To evaluate a real‐world workflow, the reference plan creation process, including image import, sphere structure generation, planning, and evaluation, was timed for each in silico case. These steps were performed sequentially to reflect a practical clinical scenario. Detailed timing data is reported in the Results section.

### 2.3 Phase 1 adaptive workflow

The adaptive portion of the workflow was simulated as a full on‐couch adaptation session using an Ethos emulator delivery environment. In a clinical setting, the patient would be positioned on the treatment couch, and a HyperSight CBCT would be acquired to capture the anatomy of the day. For this study, a previously acquired CBCT scan served as a surrogate for this adaptive imaging step.

OAR structures were generated using Ethos’ AI‐based auto‐segmentation tools and reviewed by a qualified medical physicist. The pre‐generated lattice of high‐dose spheres from the reference plan was rigidly propagated onto the daily image and aligned with the physician‐defined GTV. Rigid propagation was used to preserve the geometric integrity of the sphere matrix, maintaining consistent spacing, pattern symmetry, and dose distribution within the target.

**FIGURE 2:**
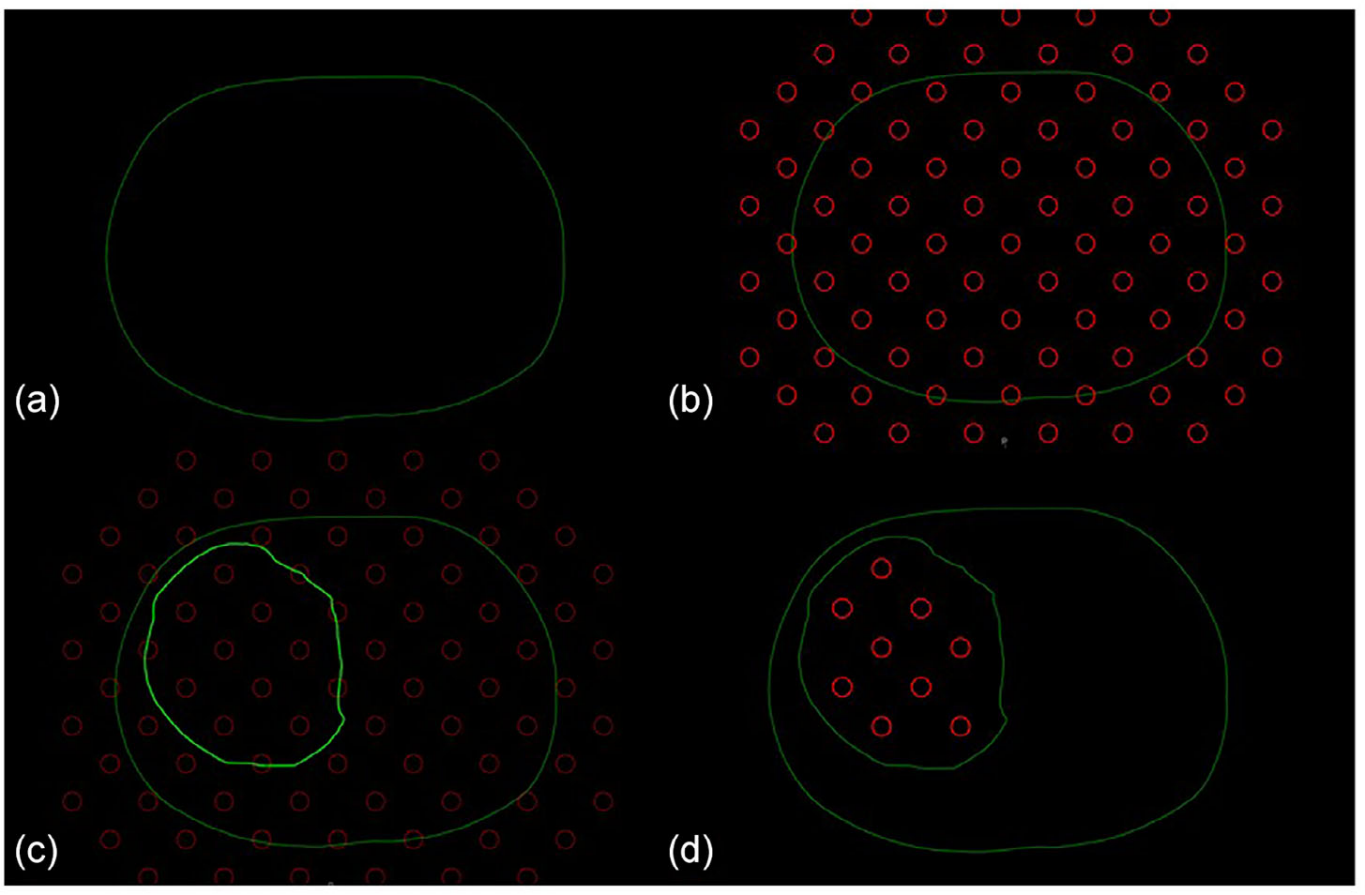
Schematic representation of the SF^2^‐ART high‐dose sphere alignment process. (a) Initial body surface; (b) uniform 3D lattice of candidate spheres overlaid on the region of the target; (c) gross tumor volume (GTV) delineation used to guide cropping; (d) final high‐dose sphere selection after exclusion of spheres within 1 cm of the GTV boundary. CT window set to all black for enhanced visualization of spheres.

For this study, the GTV was manually recreated by the research team based on contours from the original clinically delivered plan, using the planning CT as reference. The accuracy of the recreated GTV was verified by comparing it to the physician‐delineated GTV using Dice similarity coefficients and mean surface distance (MSD), with all metrics demonstrating strong agreement (Dice > 0.95, MSD < 2 mm). In a clinical scenario, this volume would be delineated by the treating physician. To ensure central placement of high‐dose spheres, all lattice components located outside the GTV or within 1 cm of its boundary were automatically removed. Any spheres that were partially cropped during this derivation were manually deleted during the review process. All structures, including the recreated GTV, were reviewed by a qualified medical physicist. However, in a clinical implementation, the adaptive workflow would involve collaborative review and approval by both the physician and medical physicist, consistent with the high‐dose, stereotactic nature of SFRT. Figure 2 shows this process of GTV delineation, sphere propagation, sphere editing, and the final PTV_High targets on an example abdominal case.

Once target review was completed, all derived structures were accepted, and the adaptive plan was generated using the predefined planning template. Optimization was performed by the Ethos IOE, and the resulting adaptive treatment plan was evaluated. The simulation‐free workflow was considered successful if the adapted plan generated correctly and met predefined clinical and dosimetric objectives equivalent to those of the clinically delivered plans. Adaptive plan quality served as the primary dosimetric endpoint, reflecting the intended “adaptive‐or‐nothing” clinical paradigm, treatment would proceed only if the adapted plan met all defined clinical goals. The duration of each step in the workflow was recorded for each patient to evaluate real‐time feasibility, including matrix Generation, pre‐plan setup, adaptive contour creation and review, and adaptive plan optimization.

### 2.4 Phase 2 workflow

Following the delivery of the Phase 1 lattice plan, patients received a second treatment phase consisting of four fractions targeting the PTV_Low with 4 Gy per fraction. Throughout this manuscript, PTV_Low denotes the uniform low‐dose target derived from the GTV via a 3–5 mm expansion (Table 1); thus, Phase 2 prescriptions are consistently referenced to PTV_Low. In this workflow, the HyperSight CBCT acquired during the Phase 1 session can be utilized as the planning image for Phase 2, thereby eliminating the need for additional simulation or image acquisition. Phase 2 delivers uniform dose to a single target volume without the complexity of lattice‐based dose sculpting, so the adaptive planning process is less intensive. A standard Ethos adaptive workflow can be applied, utilizing AI‐generated contours and the Intelligent Optimization Engine for plan generation.

### 2.5 Plan quality evaluation

Plan quality was assessed by comparing each SF^2^‐ART plan to the corresponding clinical plan that had been previously delivered. Both plans used the same dose prescription and clinical objectives. Plan evaluation focused on target coverage, dose distribution, and overall deliverability. All plans were generated and evaluated using SBRT planning constraints with tight gradients and conformity and high plan heterogeneity. The following metrics were used for quantitative comparison between the simulation‐free adaptive and clinically delivered plans to determine clinical equivalence:

- **PTV_High**: D0.03cc, V100%, Dmean, RTOG Conformity Index (CI) and V50%
- **PTV_Low**: D0.03cc, V100%, Dmean, V12Gy, and V8Gy
- **Normal Tissue** (defined as PTV_Low + 3 cm—PTV_Low): Dmax0.03cc

Organ‐at‐risk constraints were determined per clinical judgment on a case‐by‐case basis. Total monitor units (MU) were also recorded and compared to those from the clinically delivered plans. Simulation‐free adaptive plans were considered successful if the generated adaptive plans were deemed clinically equivalent or better for all dosimetric goals, were deliverable, and met standard QA metrics.

Secondary dose verification was performed on all the SF^2^‐ART adaptive plans using Mobius3D (Varian Medical Systems, Palo Alto, CA), employing a 5% dose difference and 3 mm distance‐to‐agreement gamma analysis criterion.^16^ This independent calculation served as a quality assurance measure to confirm the accuracy of the generated treatment plans.^17^

### 2.6 Workflow timing and feasibility

The end‐to‐end planning process, including image selection, lattice generation, reference plan creation, and adaptive planning and delivery, was performed for each patient without a simulation CT. Brief definitions of each workflow step are provided below:

- **Image Selection and Lattice Generation**: Reviewing patient imaging and generating a lattice matrix of high‐dose spheres. This time does not include the time that would be needed to create and acquired an image on HyperSight scan if the diagnostic CT was not usable.
- **Reference Plan Creation**: Generating and approving the Intent and initial reference plan using the predefined template.
- **Adaptive Contouring**: Evaluating and finalizing adaptive contours on‐session, including physician‐drawn GTV definition and propagation/review of the high‐dose sphere matrix.
- **Plan Optimization and Calculation**: Time taken by the Intelligent Optimization Engine (IOE) to optimize and compute the adaptive plan.
- **Total Adaptive Treatment Time**: The reported total treatment time as designed by the Ethos emulator. It is important to note this does not include patient setup, as these were done in‐silico.
- **Estimated Workflow Time**: Estimated workflow time was defined as the complete process from initial image review through delivery. This estimate includes reasonable clinical allowances for physician consultation (30 min), physics review of the intent and associated safety checks (30 min), reference plan review and delivery preparation (20 min), patient setup and CBCT acquisition (10 min), secondary dose calculation (10 min), and beam delivery time (10 min).

## 3 RESULTS

All ten cases were successfully replanned and evaluated using the proposed workflow. The results below summarize workflow timing, dosimetric comparison, and overall deliverability, with a specific focus on Phase 1 planning due to its greater complexity and reduced treatment timeline.

### 3.1 Workflow timing and feasibility

To assess the efficiency of the SF^2^‐ART workflow, each patient case was analyzed for timing across key planning and adaptive steps. All timing measurements were recorded manually by the research team using time stamps documented at each planning and adaptive step, consistent with standard clinical QA processes. Table 2 presents a detailed breakdown of the workflow, subdivided into six primary evaluation components that mirror the steps outlined in Section 2.6.

**TABLE 2:** Timing of key workflow steps.

Patient	Image selection and lattice generation (min)	Reference plan creation (min)	Adaptive contouring (min)	Target review and modification (min)	Plan optimization and calculation (min)	Simulated adaptive treatment (min)	Total estimated patient (min)
1	12	29	5.6	14.2	6.4	29.2	180.2
2	4	19	3.2	8.5	7.3	22.1	155.1
3	8	28	2.5	13.5	6.5	25.4	171.4
4	4	18	3.4	6.2	5.5	18.7	150.7
5	8	21	4.1	9.4	8.7	25.2	164.2
6	10	21	4.5	11.2	6.2	24.9	165.9
7	11	22	4.7	12.4	7.7	27.8	170.8
8	10	26	3.8	10.1	7.1	24.1	170.1
9	9	21	3.4	7.2	5.2	18.8	158.8
10	7	24	5.2	9.1	8.5	25.8	166.8
**Median**	**8.5**	**21.5**	**4.0**	**9.8**	**6.8**	**25.1**	**166.4**

Although this study was conducted in‐silico, on‐table time was estimated based on adaptive planning and delivery steps recorded in the Ethos emulator. However, patient setup and imaging acquisition times were not included, as these are expected to vary in a clinical environment. A more detailed analysis of on‐table time implications is provided in Section 4.

### 3.2 Dosimetric comparison and plan quality

Table 3 summarizes the average, minimum, and maximum absolute differences in dosimetric metrics between the clinically delivered plans and the SF^2^‐ART plans for Phase 1.

**TABLE 3:** Phase 1 evaluation metrics.

	Metric	Average Δ	Min Δ	Max Δ
PTV_High	Dmax0.03cc (Gy)	0.16	0.03	0.28
	V12Gy (%)	4.00	0.20	6.60
	Dmean (Gy)	0.19	0.02	0.51
	V6Gy (%)	0.47	0.00	1.10
	Conformity Index	0.05	0.00	0.20
PTV_Low	Dmax0.03cc (Gy)	0.37	0.02	0.83
	V4Gy(%)	2.56	0.00	7.00
	Dmean (Gy)	0.10	0.02	0.23
	V12Gy (%)	0.00	0.00	0.00
	V8Gy (%)	1.17	0.30	2.50
Normal tissue	Dmax0.03cc (Gy)	0.34	0.02	1.09
Monitor units	MU difference	235	20	1308

All plans were qualitatively reviewed and found to meet clinical expectations. Table 4 summarizes the average, minimum, and maximum differences in key dosimetric metrics between the simulation‐free and clinically delivered Phase 2 plans. As Phase 2 targets contain only PTV_Low, the evaluation was limited to PTV_Low coverage, normal tissue dose, and monitor unit comparisons. Key adaptive timing metrics for Phase 2 were also recorded and are included below.

**TABLE 4:** Phase 2 evaluation metrics between.

Structure	Metric	Average Δ	Min Δ	Max Δ
PTV_Low	Dmax0.03cc (Gy)	0.21	0.03	0.76
	V4Gy (%)	2.21	0	4.52
	Dmean (Gy)	0.14	0.04	0.26
Normal tissue	Dmax0.03cc (Gy)	0.32	0.04	0.92
Monitor units	Difference	150	12	851

	Average	Min	Max
Adaptive optimization and calculation time (min)	3.6	2.8	5.1
Simulated adaptive treatment time per session (min)	15.5	13.5	24.4

**FIGURE 3:**
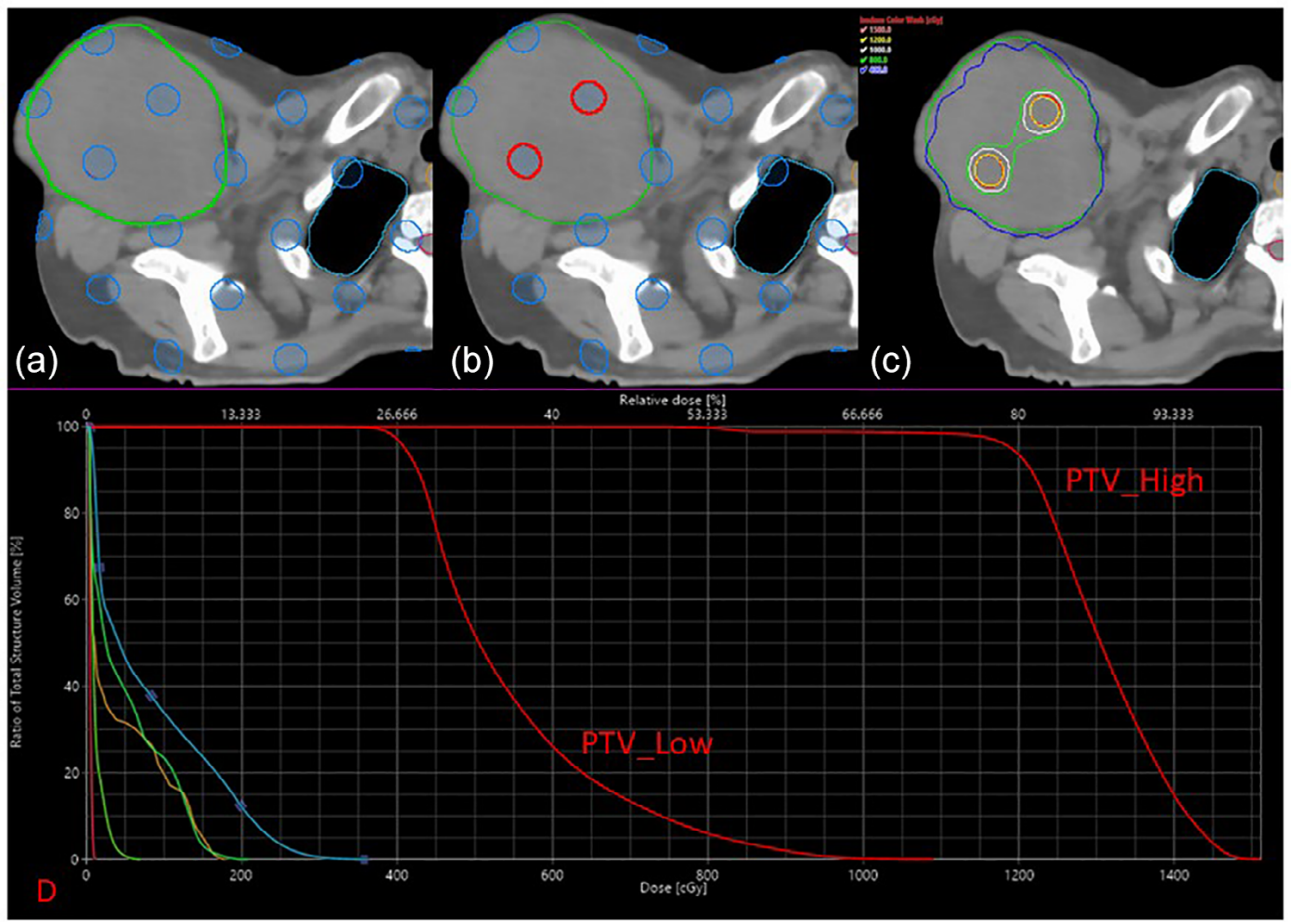
Representative patient example comparing simulation‐free SF^2^‐ART and clinically delivered plans. (a) Original lattice matrix of high‐dose spheres; (b) high‐dose spheres after cropping to remove overlaps within 1 cm of the GTV boundary; (c) isodose distribution; (d) dose–volume histogram (DVH).

The SF^2^‐ART planning process and dose distribution are depicted for a representative case in Figure 3. This illustration highlights the generated high‐dose sphere lattice, the final PTV_High structure derived from the GTV with a 1 cm retraction, and the resulting dose distribution. The corresponding dose‐volume histogram (DVH) is also shown, comparing coverage for the PTV_High and PTV_Low structures.

### 3.3 Quality assurance and deliverability

All plans (100%) passed secondary dose verification using Mobius3D (Varian Medical Systems, Palo Alto, CA), with 3D gamma pass rates exceeding 90% under 5%/3 mm criteria and a 10% threshold. Additionally, all recreated GTVs were verified to match the original physician‐defined GTVs using Dice similarity coefficient (0.95) and MSD (2 mm). The generated lattice structures were manually reviewed by the team to ensure geometric integrity after cropping, and all final plans underwent a technical deliverability assessment in the Ethos emulator. No issues were encountered during plan generation or virtual delivery, suggesting technical deliverability is consistent with current clinical standards. Together, these findings support the clinical feasibility of a simulation‐free SF^2^‐ART workflow that maintains high plan quality while significantly reducing overall treatment package time.

## 4 DISCUSSION

Simulation‐free adaptive workflows have been successfully implemented in various clinical settings, particularly in palliative and emergent treatment contexts.^7^, ^9^, ^12^ Previous reports have shown that imaging‐only workflows using either diagnostic CTs or image‐only CBCTs can safely support adaptive planning, especially when combined with quality assurance and contouring standards.^8^, ^11^ Our study demonstrates the feasibility of a simulation‐free spatially fractionated adaptive radiotherapy workflow conducted entirely in silico. In this context, in silico studies, such as ours, play a key role in mitigating risk when transitioning between workflows and informing future prospective clinical protocols.^12^, ^18^ While our analysis was conducted entirely in a virtual environment using the Ethos emulator, the findings provide an integral first step toward clinical implementation.

The intent of this study was not to evaluate the clinical efficacy of SFRT itself, as this has been previously demonstrated.^19^, ^20^, ^21^ Rather, to demonstrate that SFRT, when integrated with online adaptive workflows, can be performed safely and rapidly without the need for a simulation‐based CT, making it more accessible to patients with urgent treatment needs. One operational challenge is the generation and verification of a reference plan without traditional patient specific quality assurance. In current practice, adapted plans undergo patient specific quality assurance PSQA prior to clinical use. However, for a simulation‐free reference plan, pre‐treatment measurement may not be available. Institutions considering clinical implementation of this workflow would need to develop policies for reference plan QA and billing compliance, potentially following similar protocols used for adapted plans under existing adaptive therapy guidelines. This challenge has been addressed in the literature, which outlines recommendations for QA of simulation‐free reference plans.^22^, ^23^, ^24^

There are also inherent limitations to this study. The evaluation was limited to ten patients, and while these included a mix of thoracic and extremity cases, broader anatomical diversity and larger sample sizes would provide more generalizable insights. Additionally, because all evaluations were performed in silico, actual clinical workflow dynamics, such as contouring time variability, patient specific issues, and image quality challenges, may introduce unforeseen complexities and additional time. While the SF^2^‐ART workflow was successfully completed for all patients, several practical considerations emerged that may influence clinical implementation. In some cases, GTVs located adjacent to critical structures may require manual editing of the sphere matrix to minimize overlap with OARs. Smaller or irregularly shaped tumors may result in the deletion of most spheres after applying the 1 cm retraction margin, limiting lattice coverage. Additionally, although AI contouring has been shown to be effective for normal tissues, occasional artifacts or poor soft tissue contrast on CBCT may necessitate planning adjustments.^14^, ^25^ All of the potential complications can have planning and delivery timing implications. These potential issues highlight the importance of physician and physicist oversight, and they may inform refinement of the planning template or adaptation rules in future prospective use.

Despite these limitations, our findings support the potential of SF^2^‐ART to reduce time to initiation of treatment while maintaining dosimetric quality and safety. Importantly, in our cohort, the average time from consultation to treatment was approximately 11 days, highlighting the opportunity to reduce this interval to less than four hours with the proposed workflow, a clinically meaningful improvement. Future work should focus on prospective clinical validation, including real‐world implementation across diverse anatomical sites and integration into adaptive SBRT programs. This work can serve as a model for how emerging SBRT paradigms can be rapidly evaluated through in‐silico trials, accelerating translation into clinical environments.

## 5 CONCLUSION

This in‐silico trial demonstrates the feasibility of delivering spatially fractionated stereotactic radiotherapy using a same‐day, simulation‐free adaptive workflow. By leveraging on‐board CBCT imaging, automated structure generation, and a templated planning approach, the SF^2^‐ART workflow enables high‐quality treatment delivery without simulation‐based CT. All cases met predefined clinical and dosimetric objectives, and workflow timing supports integration into urgent or time‐sensitive care pathways. These findings establish a foundation for future clinical implementation and support continued exploration of SFRT within adaptive SBRT frameworks. The proposed workflow could enable consult‐to‐treatment planning in a single visit.

## AUTHOR CONTRIBUTIONS

*Study conception and design*: Dennis N. Stanley, Carlos E. Cardenas, and Joseph Harms. *Data acquisition*: Dennis N. Stanley, Alyssa Birchmeier, Carlos E. Cardenas, and Courtney Stanley. *Analysis and interpretation of data*: Dennis N. Stanley, Alyssa Birchmeier, Carlos E. Cardenas, Natalie Viscariello, Joel Pogue, and Joseph Harms. *Clinical oversight and interpretation*: Mehran Yusuf, Michael Soike, Samuel Marcrom, and Richard Popple. *Manuscript preparation and critical review*: Dennis N. Stanley, Alyssa Birchmeier, Carlos E. Cardenas, Natalie Viscariello, Joel Pogue, Courtney Stanley, Joseph Harms, Richard Popple, Mehran Yusuf, Michael Soike, and Samuel Marcrom. All authors reviewed and approved the final manuscript and agree to be accountable for all aspects of the work.

## CONFLICT OF INTEREST STATEMENT

Dennis N. Stanley receives consulting fees, payment or honoraria for lectures, presentations, speaker bureaus, manuscript writing, or educational events, and support for attending meetings and/or travel from Varian Medical Systems as an Educational Consultant. Richard A. Popple has contracts and honoraria for presentations with Varian Medical Systems and receives royalties for a patent licensed with UAB Research Foundation to Varian Medical Systems. The other authors declare that they have no known competing financial interests or personal relationships that could have appeared to influence the work reported in this paper.

## DECLARATION OF GENERATIVE AI AND AI‐ASSISTED TECHNOLOGIES IN THE WRITING PROCESS

During the preparation of this work, the author(s) used ChatGPT in order to proofread. After using this tool/service, the author(s) reviewed and edited the content as needed and take(s) full responsibility for the content of the publication.

## Data Availability

Research data are stored in an institutional repository and will be shared upon request to the corresponding author.
